# Supplementary material for: Exploring the Potential of the Model Cyanobacteria Synechococcus PCC 7002 and PCC 7942 for the Photoproduction of High-Value Terpenes: A Comparison with Synechocystis PCC 6803
Source: Biomolecules. 2023 Mar 9;13(3):504. doi: 10.3390/biom13030504 (PMC10046388; doi:10.3390/biom13030504)
Supplement: Supplementary file 1 [file biomolecules-13-00504-s001.zip › Figure S2.pdf]

A

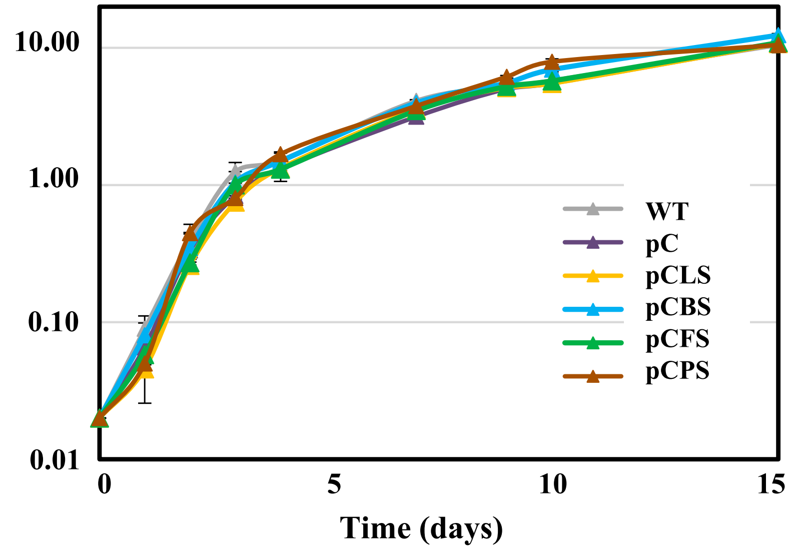

B

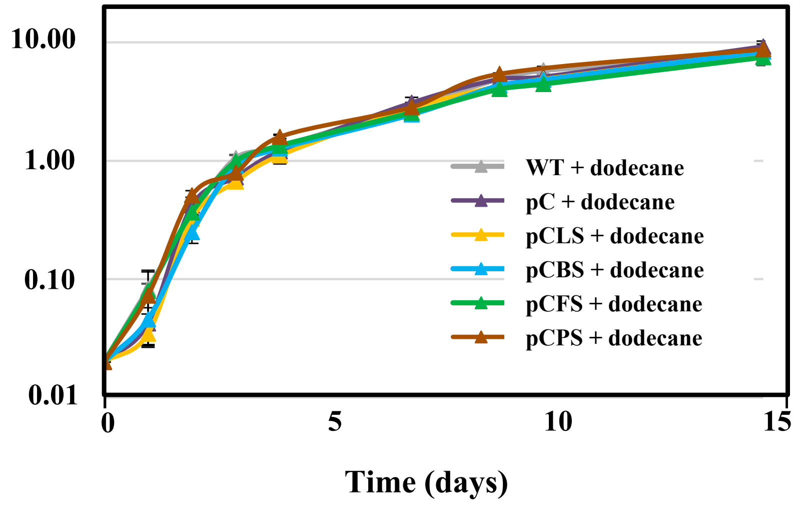

Figure S2. Influence of dodecane and pC-derived plasmids on the photoautotrophic growth of of *Synechococcus* PCC 7002

All strains were cultivated under standard light (2500 lux) and temperature (30°C) in absence (**A**) or presence (**B**) of a dodecane overlay: wild-type (**WT**), or its derivatives carrying the **empty pC vector**, or a pC-derived plasmid expressing the limonene synthase (**pCLS**), bisabolene synthase (**pCBS**), farnesene synthase (**pCFS**) or pinene synthase (**pCPS**). Errors bars represent standard deviation from biological triplicates.
