# Supplementary material for: Exploring the Potential of the Model Cyanobacteria Synechococcus PCC 7002 and PCC 7942 for the Photoproduction of High-Value Terpenes: A Comparison with Synechocystis PCC 6803
Source: Biomolecules. 2023 Mar 9;13(3):504. doi: 10.3390/biom13030504 (PMC10046388; doi:10.3390/biom13030504)

Figure S3A. GC–MS analyses of the dodecane overlays of cultures of the *Synechoccocus* PCC 7002 strains harboring the plasmids pC, pCBS or pCFS

Ion chromatograms (left panels) and corresponding mass spectra (right panels) of farnesene-isomers standards containing bisabolene (**a**) or dodecane samples from cultures of *Synechococcus* PCC 7002 propagating pCFS (**b**, production of α-farnesene), pCBS (**c**, E-α-bisabolene production) or pC (**d**, negative control). β-caryophyllene (retention time = 17.3 min) was used as the fixed-concentration (0.01 g/L) internal standard (IS) for quantification. The concentrations of bisabolene and farnesene were calculated using the standard curves that we have reported during our recent analysis of the production of bisabolene and farnesene by the other model cyanobacterium *Synechocystis* PCC 6803 (See Blanc-Garin *et al* 2022 Biotechnology for Biofuels and Bioproducts 15 [20] https://doi.org/10.1186/s13068-022-02211-0).

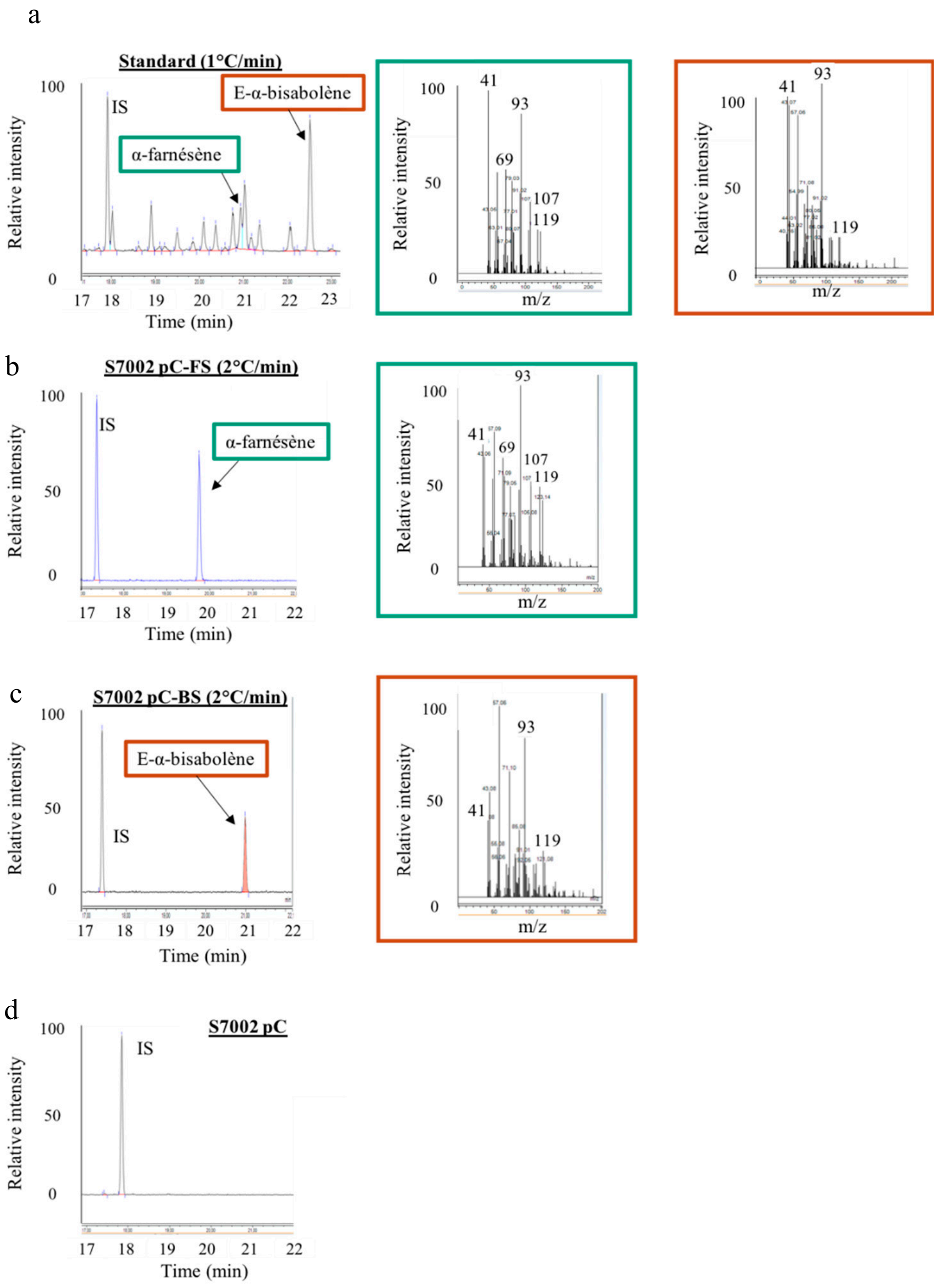

Figure S3B. GC–MS analyses of the dodecane overlay of cultures of the *Synechococcus* PCC 7002 strains harboring the plasmids pC or pCLS

Ion chromatograms (left panels) and corresponding mass spectra (right panels) of a S-(-)-limonene standard (upper panel) or dodecane samples (lower panel) of cultures of *Synechocystis* harboring either pC (negative control) or the pCLS (S-(-)-limonene production). Pinene (retention time =5.41) was used as the fixed-concentration (0.01 g/L) internal standard (IS) for quantification. The concentration of limonene was calculated using the standard curves that we employed during our recent analysis of limonene production by the other model cyanobacterium *Synechocystis* PCC 6803 (See Blanc-Garin *et al* 2022 Biotechnology for Biofuels and Bioproducts 15 [20] https://doi.org/10.1186/s13068-022-02211-0).

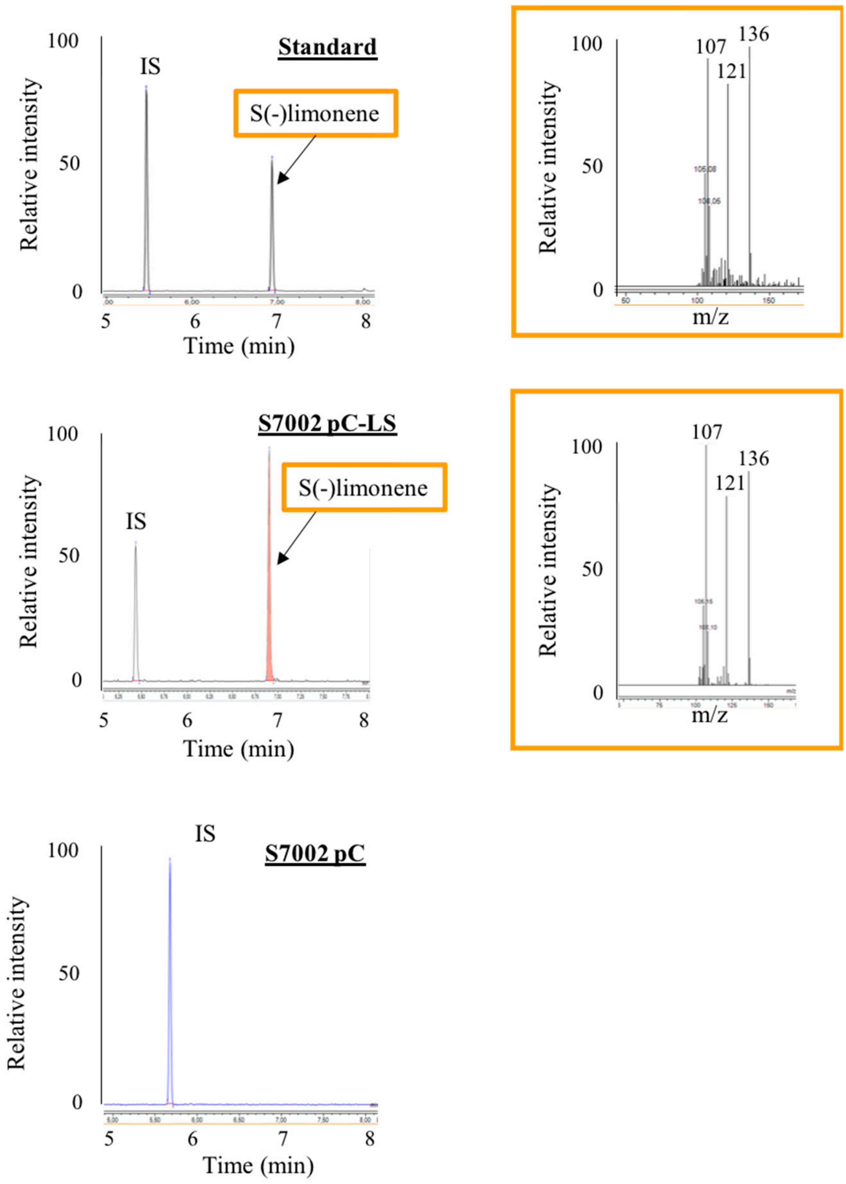

Figure S3C. GC–MS analyses of the dodecane overlay of cultures of the *Synechococcocus* PCC 7002 strains harboring the pC or pCPS plasmids

Ion chromatograms (left panels) and corresponding mass spectra (right panels) of an α-pinene standard (upper panel) or dodecane samples (lower panel) of cultures of *Synechococcus* PCC 7002 harboring either pC (negative control) or the pCPS (pinene production). S-(-)-limonene (retention time = 6.89 min) was used as the fixed-concentration (0.01 g/L) internal standard (IS) for quantification. The concentration of pinene was calculated using the standard curves that we employed during our recent analysis of pinene production by *Synechocystis* PCC 6803 (See Blanc-Garin *et al* 2022 Biotechnology for Biofuels and Bioproducts 15 [20] https://doi.org/10.1186/s13068-022-02211-0).

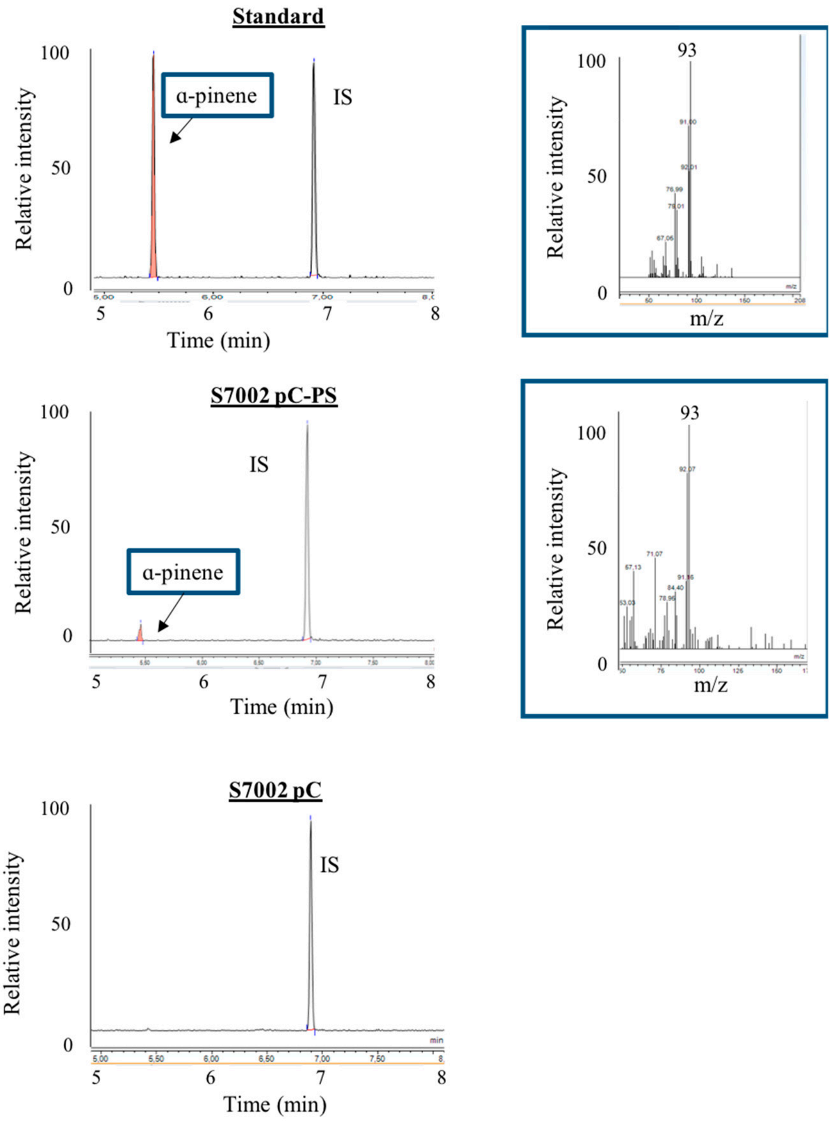

Supplement: Supplementary file 1 [file biomolecules-13-00504-s001.zip › Figure S3.pdf]
