# Supplementary material for: Exploring the Potential of the Model Cyanobacteria Synechococcus PCC 7002 and PCC 7942 for the Photoproduction of High-Value Terpenes: A Comparison with Synechocystis PCC 6803
Source: Biomolecules. 2023 Mar 9;13(3):504. doi: 10.3390/biom13030504 (PMC10046388; doi:10.3390/biom13030504)
Supplement: Supplementary file 1 [file biomolecules-13-00504-s001.zip › Figure S5.pdf]

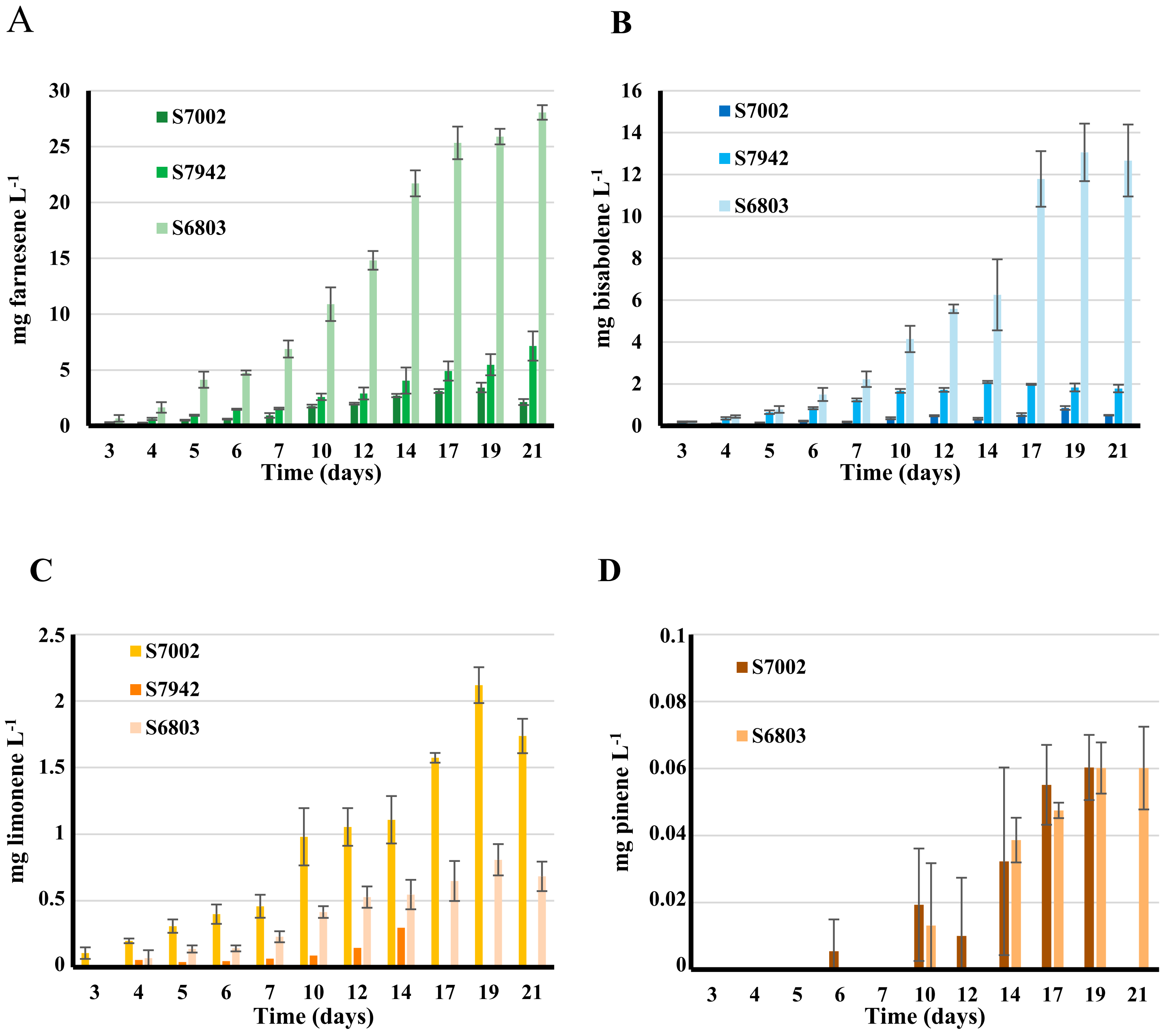

Figure S5. Comparison of the photosynthetic production of terpenes by the *Synechococcus* PCC 7002, *Synechococcus* PCC 7949 and *Synechocystis* PCC 6803 strains we have engineered in this study (S7002 and S7942), or a previous one (S6803)

Cells were grown under standard photoautotrophic conditions in the presence of a dodecane overlay (20% vol/vol) to assay the production of farnesene (**A**), Bisabolene (**B**), limonene (**C**) or Pinene (**D**) during 21 days. Error bars represent standard deviation from $n \geq 2$ biological replicates.

The terpenes producing strains of S6803 have been described in Blanc-Garin *et al* 2022 Biotechnology for Biofuels and Bioproducts 15 [20] https://doi.org/10.1186/s13068-022-02211-0.
