# Supplementary material for: Exploring the Potential of the Model Cyanobacteria Synechococcus PCC 7002 and PCC 7942 for the Photoproduction of High-Value Terpenes: A Comparison with Synechocystis PCC 6803
Source: Biomolecules. 2023 Mar 9;13(3):504. doi: 10.3390/biom13030504 (PMC10046388; doi:10.3390/biom13030504)
Supplement: Supplementary file 1 [file biomolecules-13-00504-s001.zip › Figure S6.pdf]

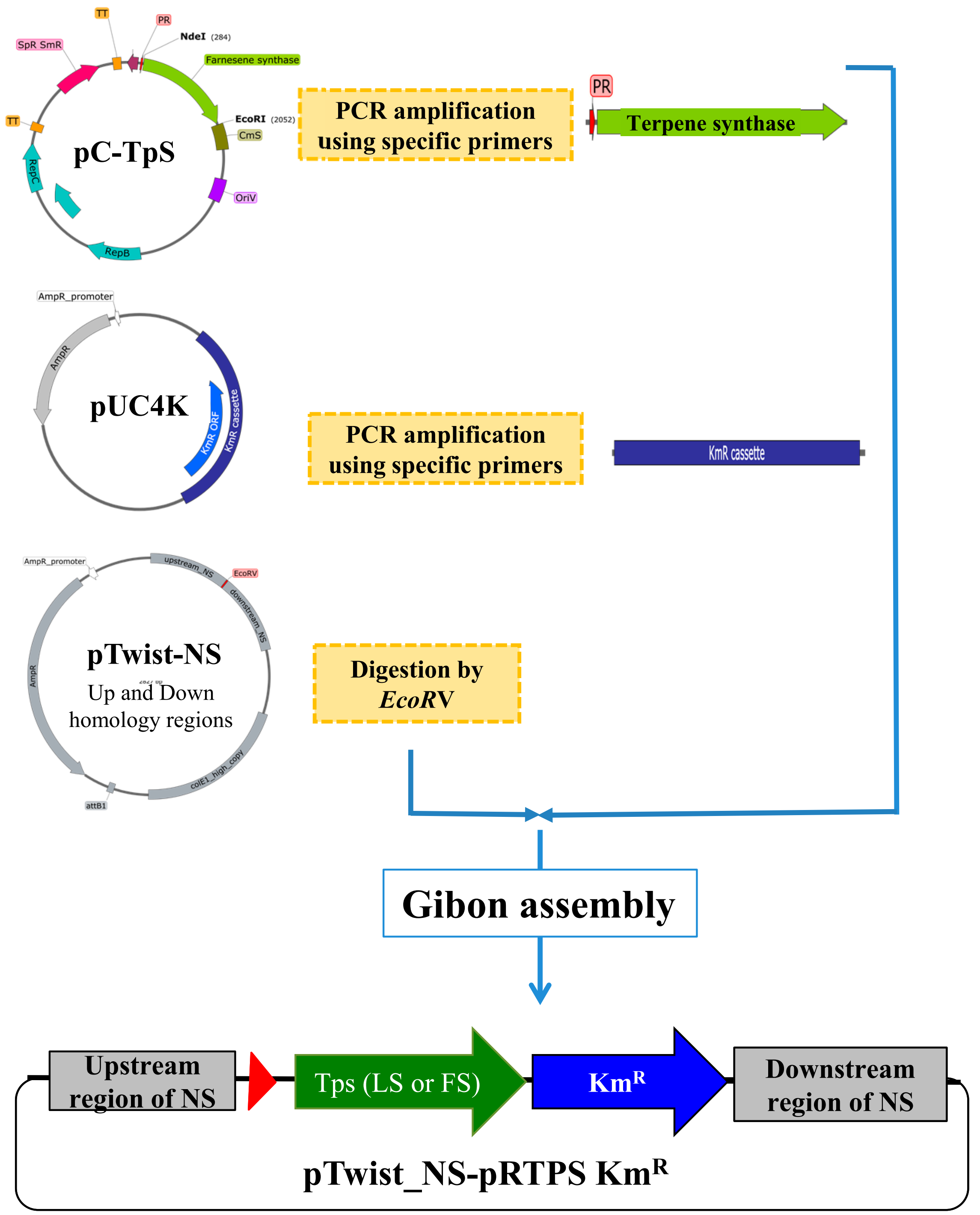

Figure S6. Construction of the pR-TPS-Km^R DNA cassette for targeted insertion of a terpene synthase genes (LS or FS) in the neutral chromosome sites of *Synechococcus* PCC 7002 (NS_{7002}) or *Synechococcus* PCC 7942 (NSI_{7942})

First, the ***pR-TPS*** gene (expression of the ***terpene synthase*** gene from the ***pR*** promoter) and the **Km^R** marker were PCR amplified from the plasmids pCTPS and pUC4K, respectively (Table S1). Second, the pTwist_NS plasmid was opened at its unique *Eco*RV restriction site flanked by the two chromosomal DNA regions surrounding the NS_{7002} or NSI_{7942} neutral sites (500 bp in length for NS_{7002} and 300 bp for NSI_{7942}). Third, all three DNA cassettes (***pR-TPS*** , **Km^R** and pTwist_NS) were assembled by Gibson® cloning. The resulting pTwist_NS_{7002}-TPS-Km^R and pTwist_NSI_{7942}-TPS-Km^R plasmids were transformed to S.7002 or S.7942 to insert the ***pR-TPS*_Km^R** cassette in the neutral chromosomic sites NS_{7002} or NSI_{7942}.
