# Supplementary material for: Exploring the Potential of the Model Cyanobacteria Synechococcus PCC 7002 and PCC 7942 for the Photoproduction of High-Value Terpenes: A Comparison with Synechocystis PCC 6803
Source: Biomolecules. 2023 Mar 9;13(3):504. doi: 10.3390/biom13030504 (PMC10046388; doi:10.3390/biom13030504)
Supplement: Supplementary file 1 [file biomolecules-13-00504-s001.zip › Figure S7.pdf]

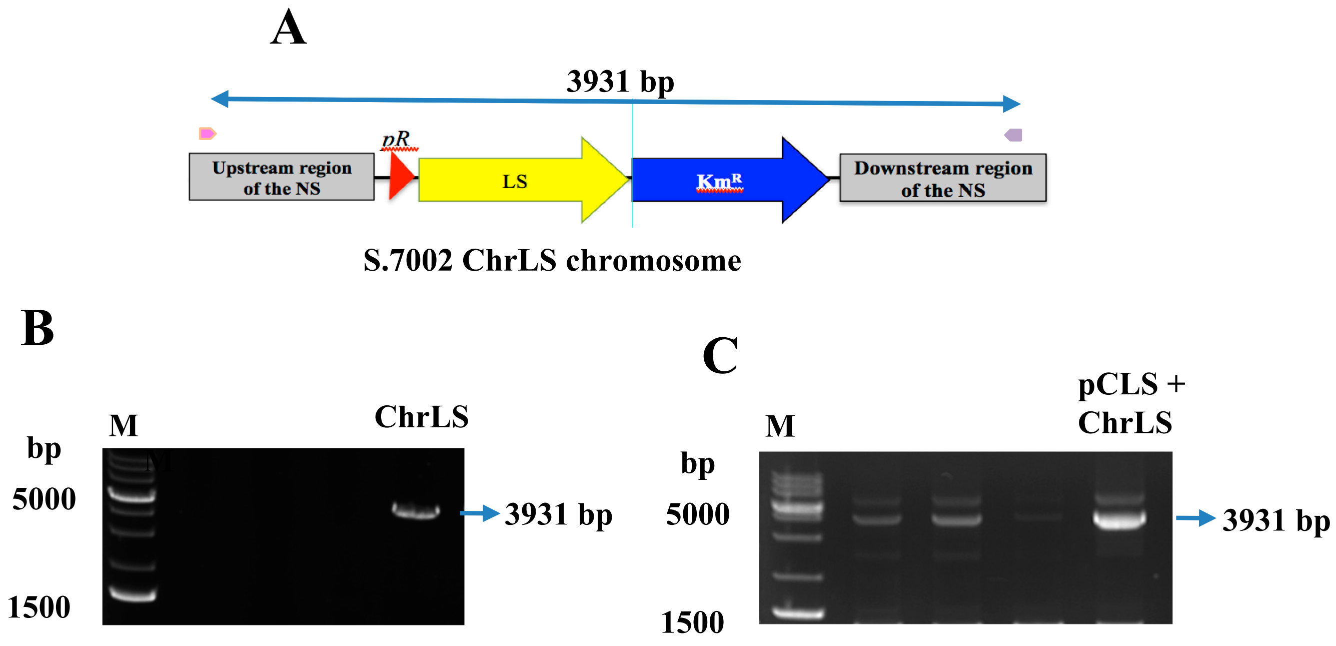


**Figure S7. PCR verification of the presence of the pR-LS-Km$^{R}$ cassette inserted inside the neutral chromosomal site (NS$_{7002}$) of *Synechococcus* PCC 7002.**

(**A**) Schematic representation of the pR-LS-Km$^{R}$ DNA cassette integrated in the NS$_{7002}$ chromosomal site of S.7002. Genes are represented by wide shapes colored in yellow (limonene synthase gene, LS), grey (NS$_{7002}$ flanking regions), blue (Km$^{R}$ marker) or red (*pR* promoter). PCR primers and the resulting 3931 bp DNA product are indicated by colored arrows.

(**B**) Typical UV-light image of the agarose gel showing the 3931 bp PCR product of the ChrLS strain harboring the pR-LS-Km$^{R}$ DNA cassette in the NS$_{7002}$ neutral chromosome site of otherwise WT cells of S.7002. Size marker (M) = Genruler 1 kb Plus DNA Ladder (Thermofisher).

(**C**) Typical 3931 bp PCR product of the pCLS+ChrLS strain harboring the pR-LS-Km$^{R}$ DNA cassette in the NS$_{7002}$ site of S.7002 cells propagating the pCLS plasmid. M = Generuler 1 kb Plus DNA Ladder (Thermofisher).
