# Supplementary material for: Exploring the Potential of the Model Cyanobacteria Synechococcus PCC 7002 and PCC 7942 for the Photoproduction of High-Value Terpenes: A Comparison with Synechocystis PCC 6803
Source: Biomolecules. 2023 Mar 9;13(3):504. doi: 10.3390/biom13030504 (PMC10046388; doi:10.3390/biom13030504)
Supplement: Supplementary file 1 [file biomolecules-13-00504-s001.zip › Figure S8.pdf]

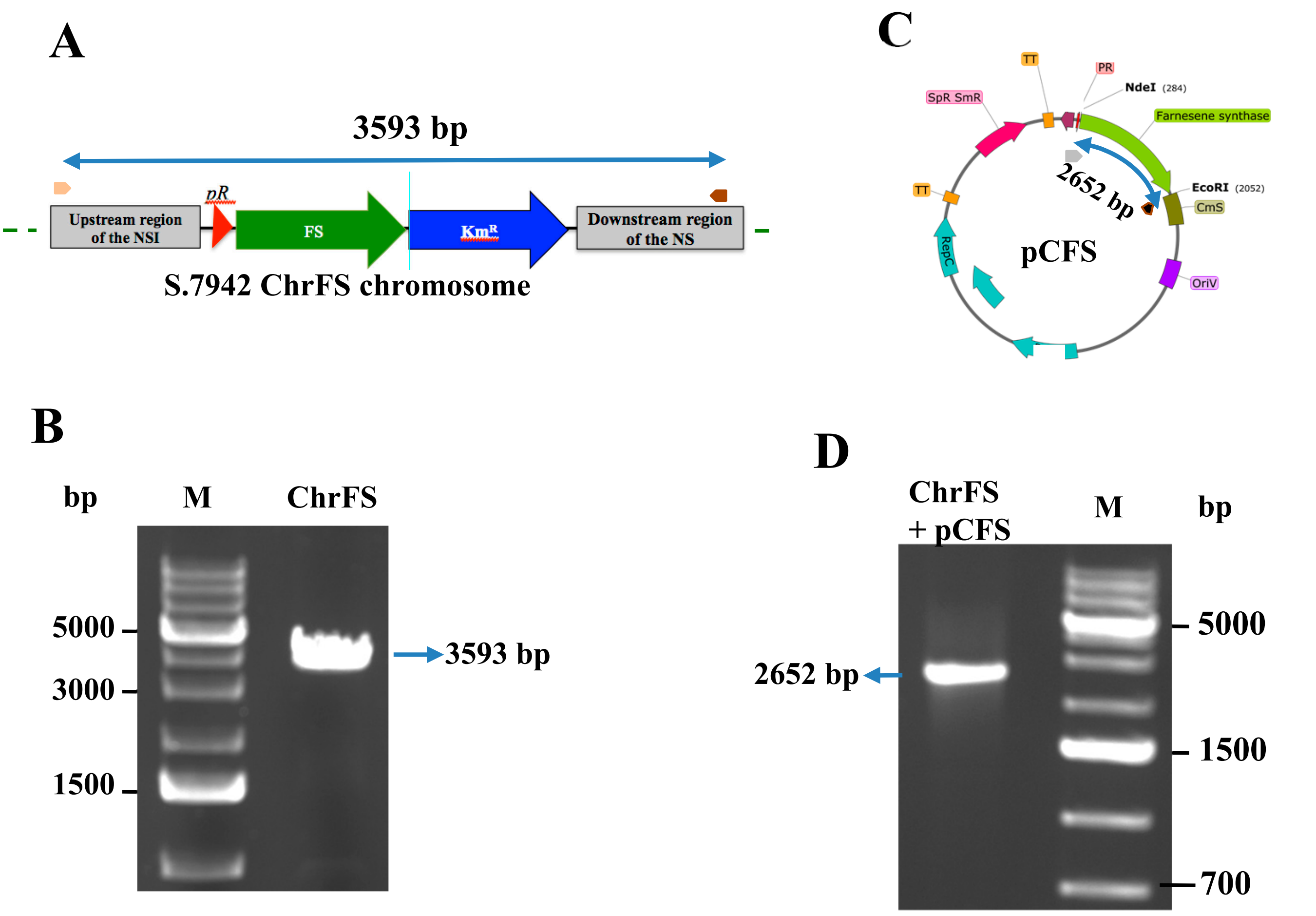

Figure S8. PCR verification of the presence of the farnesene synthase gene propagated by either or both the pCFS plasmid or the NSI_{7942} neutral chromosomal site of *Synechococcus* PCC 7942

(**A**) Schematic representation of the pR-FS-Km^R DNA cassette integrated in the NSI_{7942} chromosome site of S.7942. Genes are represented by wide shapes colored in green (farnesene synthase gene, FS), grey (NSI_{7942} flanking regions), blue (Km^R marker) or red (*pR* promoter). PCR primers and the resulting 3593 bp DNA product are indicated by colored arrows.

(**B**) Typical UV-light image of the agarose gel showing the 3593 bp PCR product of the ChrFS strain harboring the pR-FS-Km^R DNA cassette in the NSI_{7942} chromosome site of otherwise WT cells of S.7942. Size marker (M) = Genruler 1 kb Plus DNA Ladder (Thermofisher).

(**C**) Schematic representation of the pCFS plasmid showing the *pR* promoter and FS gene as the small red triangle and green arrows, respectively. PCR primers and the corresponding 2652 bp DNA product are indicated by colored arrows.

(**D**) Typical agarose gel showing the 2652 bp PCR product of the pR-FS gene of pCFS replicating in the ChrFS strain generating the ChrFS+pCFS strain. Size marker (M) = Generuler 1 kb Plus DNA Ladder (Thermofisher).
