# Supplementary material for: Exploring the Potential of the Model Cyanobacteria Synechococcus PCC 7002 and PCC 7942 for the Photoproduction of High-Value Terpenes: A Comparison with Synechocystis PCC 6803
Source: Biomolecules. 2023 Mar 9;13(3):504. doi: 10.3390/biom13030504 (PMC10046388; doi:10.3390/biom13030504)
Supplement: Supplementary file 1 [file biomolecules-13-00504-s001.zip › Table S1.pdf]

Table S1 – Characteristics of the bacterial strains and plasmids used in this study

Strain/Plasmid	Relevant features	Reference
***Escherichia coli* CM404**	*E. coli* strain harboring the self-transmissible plasmid pRK2013 enabling the conjugative transfer of RSF1010-derived pC plasmids into cyanobacteria	(Mermet-Bouvier and Chauvat, 1994)
***Escherichia coli* TOP10**	*E. coli* strain for cloning and conjugation	Invitrogen
***Escherichia coli* NEB 10-beta**	*E. coli* strain for cloning	New England Biolabs
***Synechococcus* PCC 7002**	Well studied, fast-growing unicellular and euryhaline cyanobacterium	Pasteur Institute (Paris, France)
***Synechococcus* PCC 7942**	Well studied unicellular freshwater cyanobacterium	Pasteur Institute (Paris, France)
**Plasmid cloning vectors**		
**pTwist**	Amp^R plasmid vector	Twist Biosciences
**pUC4K**	Source of the Km^R marker with no transcription terminator	Pharmacia
**pC**	RSF1010-derived plasmid vector (Sp^R/Sm^R, Cm^R) harboring the strong p_R promoter for constitutive gene expression in *E. coli* and cyanobacteria	(Veaudor *et al.*, 2018)
**Replicative plasmids for strong constitutive expression of terpene synthase genes in cyanobacteria**		
**pCLS**	pC derived plasmid (Sp^R/Sm^R, Cm^S) harboring the *Mentha spicata* 4S-limonene synthase gene (LS) cloned between the *Nde*I and *EcoR*I restriction sites	(Chenebault *et al.*, 2020) & This study
**pCPS**	pC derivative (Sp^R/Sm^R, Cm^S) harboring the *Pinus taeda* pinene synthase gene (PS) cloned between *Nde*I and *EcoR*I	(Blanc-Garin *et al.*, 2022) & This study
**pCBS**	pC derivative (Sp^R/Sm^R, Cm^S) carrying the *Abies grandis* bisabolene synthase gene (BS) cloned between *Nde*I and *EcoR*I	(Blanc-Garin *et al.*, 2022) & This study
**pCFS**	pC derivative (Sp^R/Sm^R, Cm^S) carrying the *Picea abies* farnesene synthase gene (FS) cloned between *Nde*I and *EcoR*I	(Blanc-Garin *et al.*, 2022) & This study
**Plasmids for gene cloning into the NS_{7002} neutral chromosomal site of *Synechococcus* PCC 7002**		
**pTwist_NS-S7002**	pTwist harboring an *Eco*RV restriction site flanked by two 500 bp chromosomal DNA regions for cloning genes in the NS_{7002} neutral chromosome site of *Synechococcus* PCC 7002	This study (Twist Bioscience) and Figure S7
**pTwist_NS-S7002p_RLSKm^R**	pTwist_NS-S7002 harboring the Km^R DNA cassette expressing the *Mentha spicata* 4S-limonene synthase from the strong p_R promoter	This study; Figure S7
**Plasmids for gene cloning into the NS_{7942} neutral chromosomal site of *Synechococcus* PCC 7942**		
**pTwist_NSI-S7942**	pTwist harboring an *Sma*I restriction site flanked by two 300 bp regions chromosomal DNA regions for cloning genes in the NSI_{7942} neutral chromosome site of *Synechococcus* PCC 7942	This study (Twist Bioscience) Figure S7
**pTwist_NSI-S7942-p_RFSKm^R**	pTwist_NS-S7942 harboring the Km^R DNA cassette expressing the *Picea abies* farnesene synthase gene (FS) from the strong p_R promoter	This study; Figure S8

References:

- [15] Mermet-Bouvier, P.; Chauvat, F. *Curr. Microbiol.* **1994**, *28*, 145–148, doi:10.1007/BF01571055.
- [23] Chenebault, C. *et al. Front. Microbiol.* **2020**, *11*, 586–601, doi:10.3389/fmicb.2020.586601.
- [20] Blanc-Garin, V.; Chenebault, C. *et al. Biotechnol. Biofuels* **2022**, *15*, doi:10.1186/s13068-022-02211-0.
- [24] Veaudor *et al. PLoS ONE* **2018***, 13*, e0198836.
