# Supplementary material for: Exploring the Potential of the Model Cyanobacteria Synechococcus PCC 7002 and PCC 7942 for the Photoproduction of High-Value Terpenes: A Comparison with Synechocystis PCC 6803
Source: Biomolecules. 2023 Mar 9;13(3):504. doi: 10.3390/biom13030504 (PMC10046388; doi:10.3390/biom13030504)
Supplement: Supplementary file 1 [file biomolecules-13-00504-s001.zip › Table S2.pdf]

Table S2 – Characteristics of the primers used in this study

Name	Sequence (5’-3’)	Purpose
**pC and pC derivatives**		
pC_Fw	TCATAAATTGCTTTAAGGCG	Forward (Fw) and reverse (Rv) primers for PCR amplification and DNA sequencing of the terpene synthase genes cloned in pC
pC_Rv	GTGTAACAAGGGTGAACAC	
**pTwist plasmids**		
M13_Fw	GTAAAACGACGGCCAGT	DNA sequencing of inserts cloned in pTwist
M13_Rv	CAGGAAACAGCTATGAC	
**Pinene synthase encoding gene**		
PS_Fw1	GCGAGAAGTTGCTAGAATTGGC	Forward and reverse primers for DNA sequencing of PS genes
PS_Rv1	TAACCGCAAAATGGCGCAAA	
PS_Rv2	ACCAACGAGACAGAGACTGC	
**Limonene synthase encoding gene**		
LS_Rv3	GATAGGCCCATGCGTTGAAGAT	Forward and reverse primers for DNA sequencing of LS genes
LS_Fw3	TGTCCGATTATAATGCCTCCG	
**Bisabolene synthase encoding gene**		
BS_Fw2	TGAGACCGACCAAGATAGC	Forward and reverse primers for DNA sequencing of BS genes
BS_Fw3	TAGGCATTGGAACGAGCG	
BS_Fw4	TATGACACCTACGGTACC	
BS_Rv5	CAAGAACTCAAGGCACTTC	
**Construction of the NS_{7002}::p*R*-LS-Km^R and NSI_{7942}::p*R*-FS-Km^R DNA cassettes by GIBSON assembly**		
Pr-fwd-S7942	gacgagcagggactcgacccTTGACTATTTTACCTCTGGC	Forward primer for PCR amplification of the *pR*-FS DNA cassette of the pCFS plasmid. It overlaps the upstream region of the NSI_{7942} chromosomal site (in 5’) and *pR* (in 3’)
Pr-fwd-S7002	acttaaaaactgagtaagatTTGACTATTTTACCTCTGGC	Forward primer for PCR amplification of the *pR*-LS and *pR*-FS DNA cassettes of the pCLS and pCFS plasmids. It overlaps the upstream region of the NS_{7002} chromosomal site (in 5’) and *pR* (in 3’)
Km-rev-S7002	aaaacgagataaaataggatGACCTGCAGGGGGGGGGG	Reverse primer for PCR amplification of the Km^R gene of pUC4K. It overlaps the downstream region of NS_{7002} (in 5’) and the end of Km^R (in 3’)

Pr-FS-Rv	cctgcaggtcTTACATGGGAACGGGTTC	Reverse primer for PCR amplification of the *pR*-FS cassette of pCFS. It overlaps the end of the FS gene (in 3') and the beginning of the Km^R marker (in 5')
Pr-LS-Rv	cctgcaggtcTTAAGCAAAGGGCTCAAAC	Reverse primer for PCR amplification of the *pR*-LS cassette of pCLS. It overlaps the end of the LS gene (in 3') and the beginning of Km^R (in 5')
KM-FS-Fwd	tcccatgtaaGACCTGCAGGGGGGGGGG	Forward primer for PCR amplification of the Km^R gene of pUC4K. It overlaps the end of FS (in 3') and the beginning of Km^R (in 5')
KM-LS-Fwd	ctttgcttaaGACCTGCAGGGGGGGGGG	Forward primer for PCR amplification of the Km^R gene of pUC4K. It overlaps the end of LS (in 3') and the beginning of Km^R (in 5')
KM-rev-S7942	ttcctgctccagaagccccGACCTGCAGGGGGGGGGG	Reverse primer for PCR amplification of the Km^R gene of pUC4K. It overlaps the downstream region of NSI neutral site (in 5') and the end of Km^R (in 3')
**Plasmids and *Synechococcus* PCC 7002 and *Synechococcus* PCC 7942 strains harboring the DNA cassettes**		
NS_S7002_Fw	ATTGGGGAATAGTGCGAC	PCR amplification of the *pR*-LS-Km^R and *pR*-FS-Km^R DNA cassette inserted into the NS_{7002} chromosomal site of S.7002, and analysis of chromosome segregation
NS_S7002_Rv	ATGTGCTTTCAGCAAAAGTT	
NS7942_Fwd	CACAGACATCTAGATAGTCCTCAA	PCR amplification and DNA sequencing of the FS expression cassette (*pR*-FS-Km^R) cloned into the NSI_{7942} chromosomal site of S.7942, and analysis of chromosome segregation.
NS7942_Rv	CTGAAGCGGACATTGCTA	
