# Supplementary figures and images for: Exploring the Potential of the Model Cyanobacteria Synechococcus PCC 7002 and PCC 7942 for the Photoproduction of High-Value Terpenes: A Comparison with Synechocystis PCC 6803
Source: Biomolecules. 2023 Mar 9;13(3):504. doi: 10.3390/biom13030504 (PMC10046388; doi:10.3390/biom13030504)

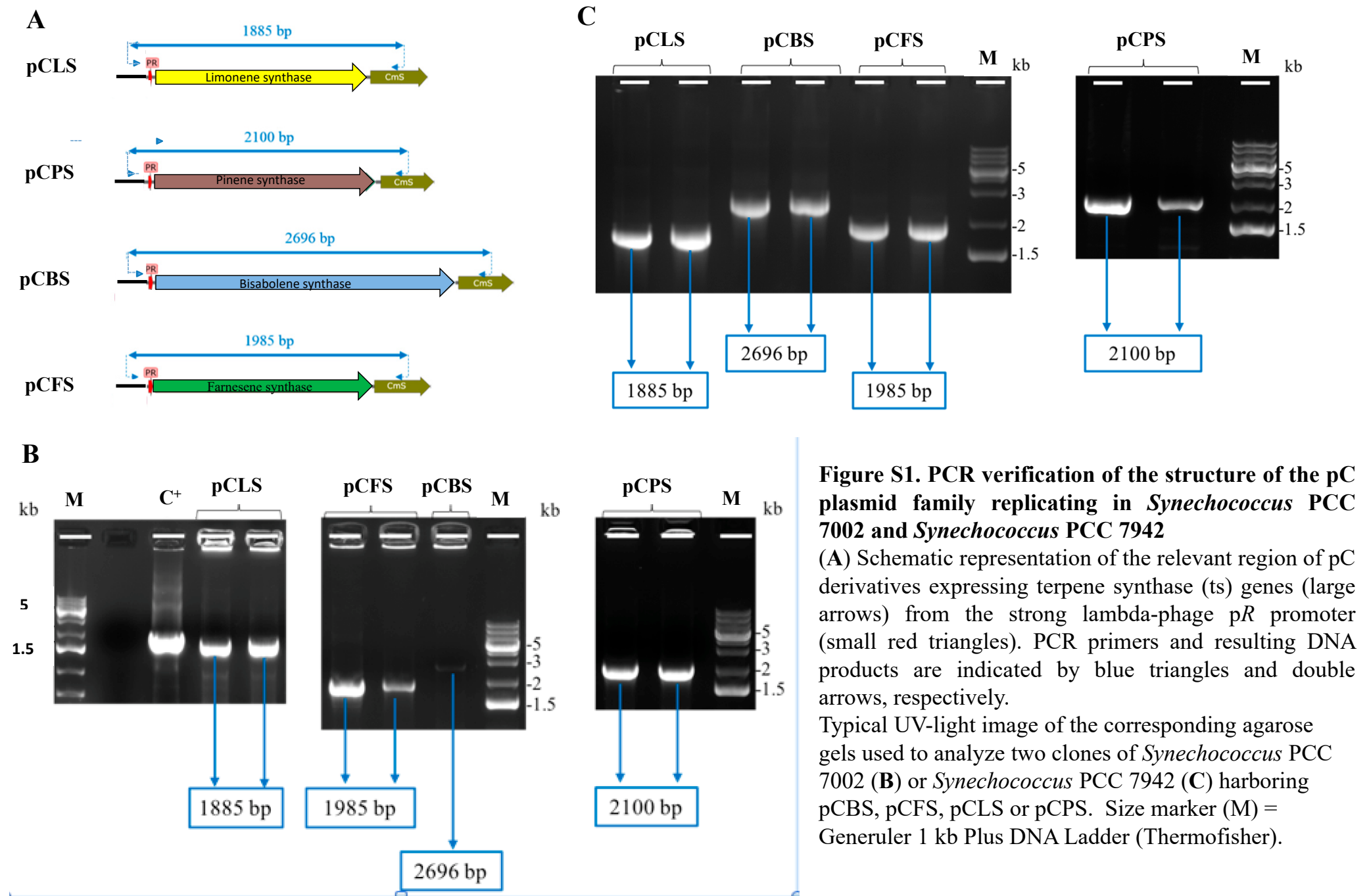

Supplement: Supplementary file 1 [file biomolecules-13-00504-s001.zip › Figure S1.pdf]
